# Supplementary material for: Foliar microbiome transplants confer disease resistance in a critically-endangered plant
Source: PeerJ. 2017 Nov 10;5:e4020. doi: 10.7717/peerj.4020 (PMC5683046; doi:10.7717/peerj.4020)
Supplement: Supplemental Information 1 — Fig S1–Community similarity vs. infection load. Fig S2–NMDS of donor and recipient communities. Fig S3–Barcharts of taxonomic profiles; Code used to generate figures from raw data; Clustal alignment of pathogen with voucher specimen; Permanova tables. Fig S4–Rarefaction curves of all samples. [file peerj-05-4020-s001.pdf]

Supporting Information for:
Total foliar microbiome transplants confer disease resistance to a critically-endangered Hawaiian endemic

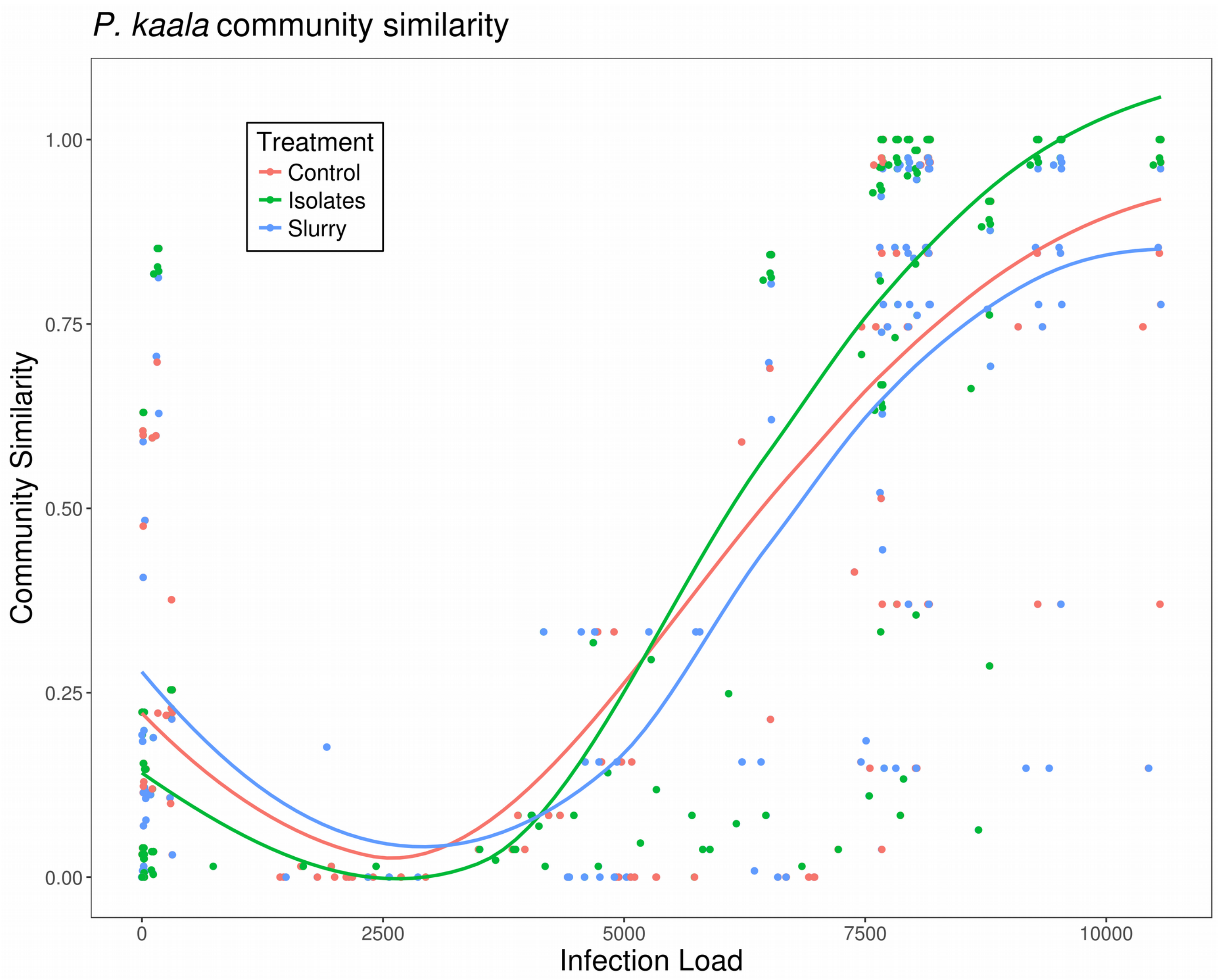

SI Figure 1) Community similarity vs. infection load

Bray-Curtis community similarity as a function of infection load similarity, showing that a major driver in community identity was *N. galeopsidis* infection rate. At very low and very high infection rates, endophytic communities were more similar. This reflects lower diversity in greenhouse-reared plants prior to treatment and the dominance of the pathogen, respectively. At intermediate infection rates, communities were more dissimilar, reflecting the establishment of inoculated communities prior to pathogen proliferation.

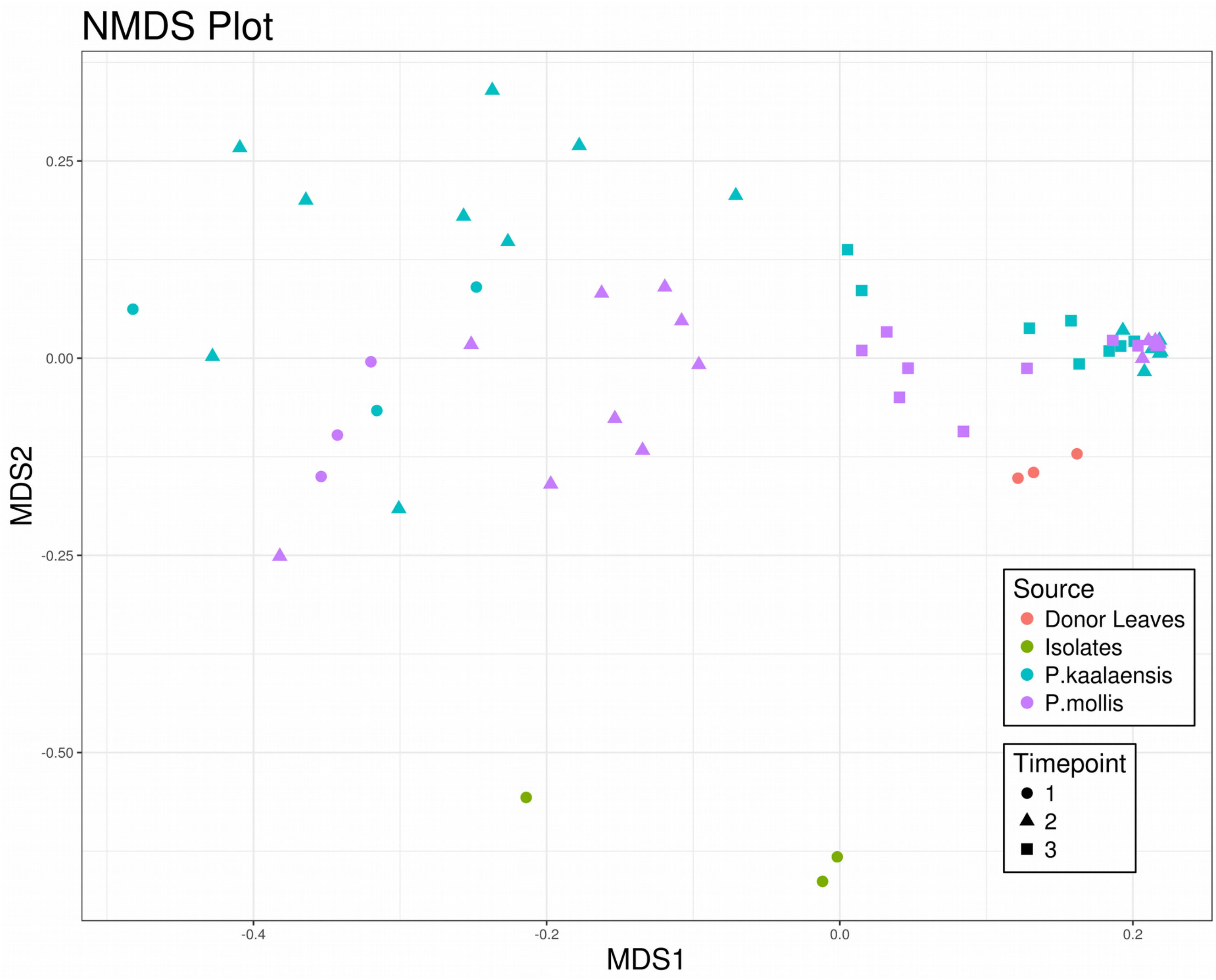

SI Figure 2) NMDS of donor and recipient communities

NMDS ordination of Bray-Curtis community similarity. Points are colored by treatment, with inoculum donors in red (*P. hirsuta* slurry) and green (fungal isolate slurry), and shapes represent sampling timepoints: Initial samples (circles), Mid-Growth samples (triangles), and Final samples (squares). Statistical analyses of Species and Timepoint are below.

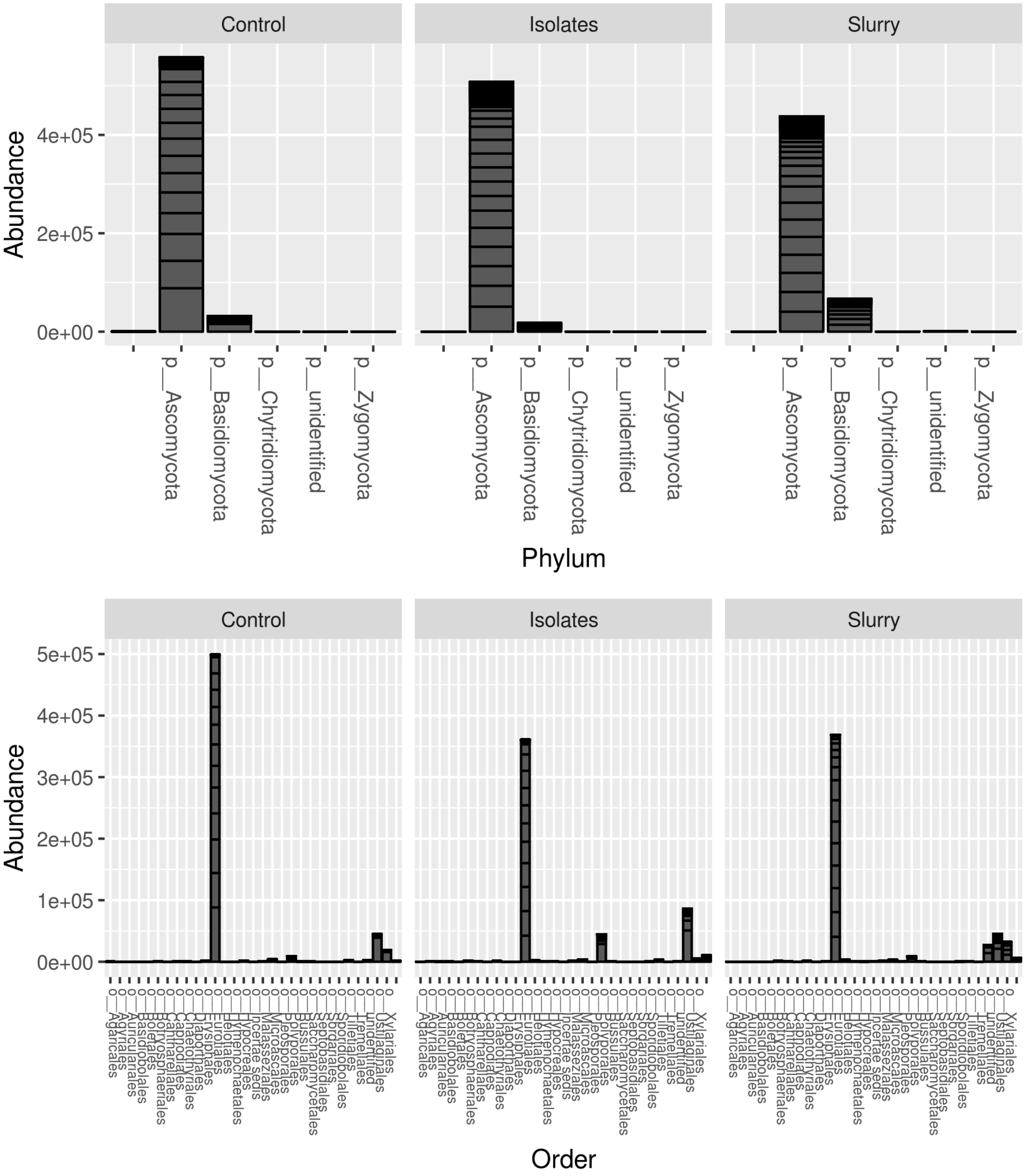

SI Figure 3) Barcharts showing taxonomic profiles (at Phylum and Order levels) of endophytic communities grouped by treatment. Measurements are the sums across all sampling periods.

Code used to create figures:

```
# Code used to generate figures - R version 3.1.2 #
# Associated data files are included in the .zip archive #


library(ggplot2)
pd = position_dodge(3)


# Figure 1


disease_data = read.csv(file = "Plant_Disease_Progress.csv", stringsAsFactors = FALSE)
disease_data$Date = as.Date(disease_data$Date)


Fig_1 = ggplot(disease_data, aes(x = Date, y = Percent.Infected, col = Group, width = 5)) +
 geom_line(position=pd, size = 1.1) +
 geom_errorbar(aes(ymin = (Percent.Infected - ci), ymax =  (Percent.Infected + ci), width = c(disease_data$wd)),
                position = pd, size = 1.1) +
 labs(title = "Phyllostegia Disease Progression", x = "Date", y = "Disease Severity", col = "Treatment") +
 theme_bw() + theme(panel.grid.major = element_blank(), panel.grid.minor = element_blank(), legend.background = element_rect(colour = "black"))+
 scale_y_continuous(breaks = c(0,.25,.5,.75,1)) + coord_cartesian(ylim = c(-.1, 1.15)) +
 facet_wrap(~ Facet, scales = "free_x") +
 scale_color_manual(values = c("#CDCDCD","#858585","#4D4D4D")) +
 theme(axis.text = element_text(size=16), axis.title = element_text(size = 18),
       legend.text = element_text(size = 14), legend.title = element_text(size = 16),
       plot.title = element_text(size = 20), strip.text.x = element_text(size = 12, face = 'bold'), legend.position = c(.615,.89))


# Figure 2


Slurry_Data <- read.csv(file = "Slurry_Taxa_data_Frame.csv")


Fig_2 = ggplot(Slurry_Data, mapping = aes(x = reorder(Species, 1/Rel_Abund), y = Rel_Abund, fill = Round)) +
 geom_bar(stat = "identity") +
 theme_bw() +
 labs(x = "UNITE Taxonomy", y = "Relative abundance", title = "Composition of Slurry Treatments", fill = "Experimental\nRound") +
 scale_fill_manual(values = gray.colors(3)) +
 facet_wrap(~ Slurry_Source, scales = "free_x") +
 theme(axis.text.x = element_text(angle=90, face = 'bold.italic', size = 12), axis.title = element_text(size = 18),
       strip.text.x = element_text(size = 12, face = 'bold'), panel.grid.major = element_blank(),
       panel.grid.minor = element_blank(), legend.background = element_rect(colour = "black"),
       legend.text = element_text(size = 14), legend.title = element_text(size = 16),
       plot.title = element_text(size = 20), legend.position = c(.9,.8))


# Figure 3


P.aphidis_v_Infection = read.csv(file = "P.aphidis_v_Infection.csv")


Fig_3 = ggplot(P.aphidis_v_Infection, mapping = aes(x = sqrt(P.aphidis_v_Infection$P.aphidis), y = P.aphidis_v_Infection$Infection_Load)) +
 geom_point() +
```

```
  stat_smooth(method = "loess", color = 'black') +
  theme_bw() +
  theme(panel.grid.major = element_blank(), panel.grid.minor = element_blank(), legend.background = element_rect(colour = "black")) +
  labs(x = expression(italic("Pseudozyma aphidis")~"abundance (sqrt)"), y = "Pathogen Infection Load") +
  theme(axis.title = element_text(size = 14)) +
  ggtitle(expression("Greater"~italic("P. aphidis")~"abundance results in lower disease severity")) +
  theme(axis.title = element_text(size = 18), plot.title = element_text(size = 20), axis.text = element_text(size = 12))

# Figure S1

PK.Infection.and.otus = read.csv(file = "Community_vs_Infection.csv")

Fig_S1 = ggplot(PK.Infection.and.otus, mapping = aes(x=jitter(PK.Infection.and.otus[,2]),
                                                      y = PK.Infection.and.otus[,1],
                                                      col = PK.Infection.and.otus[,3])) +
  geom_point() +
  geom_smooth(se = FALSE) +
  theme_bw() +
  labs(x = "Infection Load", y = "Community Similarity", colour = "Treatment") +
  ggtitle(expression(italic("P. kaala")~"community similarity")) +
  theme(panel.grid.major = element_blank(), panel.grid.minor = element_blank(), legend.background = element_rect(colour = "Black"),
        axis.title = element_text(size = 18), axis.text = element_text(size = 12), plot.title = element_text(size = 20),
        legend.text = element_text(size = 14), legend.title = element_text(size = 16), legend.position = c(.2,.85))

# Figure S2

NMDS = read.csv(file = "NMDS_Data.csv")
NMDS$Timepoint = factor(NMDS$Timepoint)

Fig_S2 = ggplot(NMDS, aes(x=MDS1, y=MDS2, col=Species, shape = Timepoint)) +
  geom_point(size = 3) +
  theme_bw() +
  labs(title = "NMDS Plot", x= "MDS1", y= "MDS2") +
  guides(col=guide_legend(title="Source")) +
  theme(legend.text = element_text(size = 14), legend.title = element_text(size = 16),
        legend.background = element_rect(colour = "black"), title = element_text(size = 20, hjust = 0.5),
        axis.title = element_text(size = 18), legend.position = c(.9,.23))
```

Clustal alignment of OTUs assigned to *N. galeopsidis* and voucher sequence from Hawaii found on *P. kaalaensis* (GenBank Accession: AB498948.1)

```
CLUSTAL O(1.2.3) multiple sequence alignment


AB498948.1_Neoerysiphe_galeopsidis           CAGAGCGTGAGGCTCTGCCCGGCTTCCCGCCGCGCGCAGAGTCGACCCTCCACCCGTGTT	60
New.ReferenceOTU120                          CATAGCTTGAGGCTCTGCCCGGCTTCCCGCCTCTCTCAGAGTCGACCCTCCACCCGTGTT	60
New.CleanUp.ReferenceOTU1041_singleton       GGGAGCGTGAGGGGGGGGGGGGCTTCCCGCCGCGCGCAGAGTCGACCCTCCACCCGTGTT	60
New.CleanUp.ReferenceOTU136_singleton        CATATCTTGAGGCTCTGCCCGGCTTCCCGCCGCGCGCAGAGTCGACCCTCCACCCGTGTT	60
New.CleanUp.ReferenceOTU154_singleton        GGGAGCGTGAGGCTGTGCCCGGCTGCCGGCCGCGCGCAGAGGGGGGCCTCCACCCGTGTT	60
New.CleanUp.ReferenceOTU320_singleton        CATAGCGTGAGGCTCTGCCCGGCTTCCCGCCGCTCTCAGAGTCGACCCTCCACCCGTGTT	60
                                                * * *****    *   **** ** *** * * *****  *  **************

AB498948.1_Neoerysiphe_galeopsidis           AACCTATATCATGTTGCTTTGGCGGATCGAGCCCTCGGCCCA-CGGCTTTTGCTGGAGCG	119
New.ReferenceOTU120                          AACCTTTATCATGTTGCTTTGGCGGATCGAGCCCTCGTCCCACCGGCTTTTGCTGGAGCG	120
New.CleanUp.ReferenceOTU1041_singleton       AACCTTTATCATGTTGCTTTGGCGGATCGAGCCCTCGGCCCACCGGCTTTTGCTGGAGCG	120
New.CleanUp.ReferenceOTU136_singleton        AACCTTTATCATGTTGATTTGGCGGATCGAGCCCTCGGCCCACCGGCTTTTGCTGGAGCG	120
New.CleanUp.ReferenceOTU154_singleton        AACCTTTATCATGTTGCTTTGGCGGATCGAGCCCTCGGCCCACCGGCTTTTGCTGGAGCG	120
New.CleanUp.ReferenceOTU320_singleton        AACCTTTATCATGTTGATTTGGCGGATCGAGCCCTCGGCCCACCGGCTTTTGCTGGAGCG	120
                                             ***** ********** *******************  **** *****************

AB498948.1_Neoerysiphe_galeopsidis           TGTCCGCCAAAGACTCAACCTAACTCGTGTAAACATGCAGTCTAAGGAAAGATTTTGAAT	179
New.ReferenceOTU120                          TGTCCGCCAAAGACTCAACCTAACTCGTGTAAACATGCAGTCTAAGGAAAGATTTTGAAT	180
New.CleanUp.ReferenceOTU_singleton           TGTCCGCCAAAGACTCAACCTAACTCGTGTAAACATGCAGTCTAAGGAAAGATTTTGAAT	180
New.CleanUp.ReferenceOTU136_singleton        TGTCCGCCAAAGACTCAAAATAACTCGTGTAAACATGAAGTCTAAGGAAAGATTTTGAAT	180
New.CleanUp.ReferenceOTU154_singleton        TGTCCGCCAAAGACTCAACCTAACTCGTGTAAACATGCAGTCTAAGGAAAGATTTTGAAT	180
New.CleanUp.ReferenceOTU320_singleton        TGTCCGCCAAAGACTCAACATAACTAGTGTAAAAATGCAGTCTAAGGAAAGATTTTGAAT	180
                                             ******************  ***** ******* *** **********************

AB498948.1_Neoerysiphe_galeopsidis           CATTA	184
New.ReferenceOTU120                          AATTA	185
New.CleanUp.ReferenceOTU1041_singleton       CATTA	185
New.CleanUp.ReferenceOTU136_singleton        AATTA	185
New.CleanUp.ReferenceOTU154_singleton        CATTA	185
New.CleanUp.ReferenceOTU320_singleton        CATTA	185
                                              ****
```

Permanova results for community distance matrix:

PLANT SPECIES

```
adonis(otus_dist ~ Species)


Permutation: free
Number of permutations: 999

Terms added sequentially (first to last)

          Df SumsOfSqs MeanSqs F.Model      R2 Pr(>F)
Species    3    1.4645 0.48818  1.7158 0.07901  0.063
Residuals 60   17.0712 0.28452         0.92099
Total     63   18.5358                 1.00000
```

TIMEPOINT

```
adonis(otus_dist ~ Timepoint)


Permutation: free
Number of permutations: 999

Terms added sequentially (first to last)

          Df SumsOfSqs MeanSqs F.Model      R2 Pr(>F)
Timepoint  1    2.2945 2.29446  8.7589 0.12379  0.001
Residuals 62   16.2413 0.26196         0.87621
Total     63   18.5358                 1.00000
```

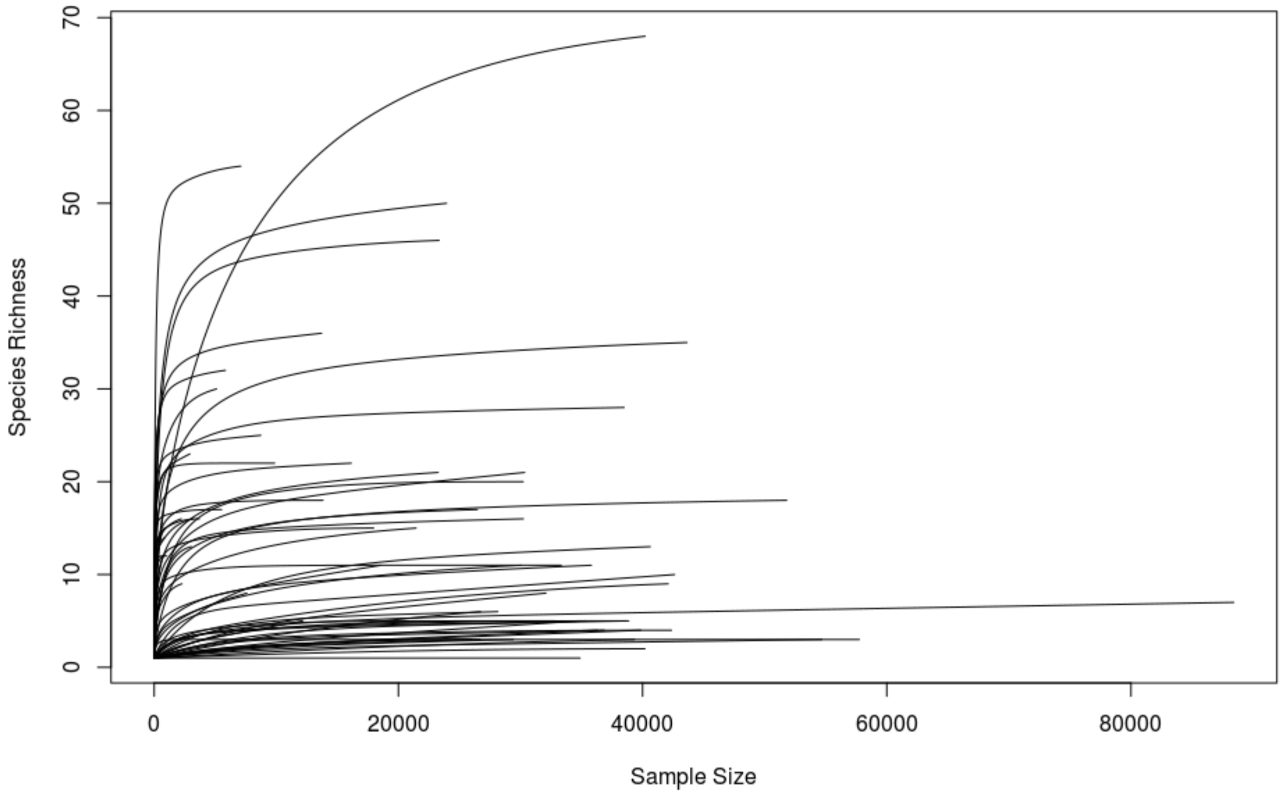

SI Figure 4) *Rarefaction curves of all samples*
