# Supplementary material for: Foliar microbiome transplants confer disease resistance in a critically-endangered plant
Source: PeerJ. 2017 Nov 10;5:e4020. doi: 10.7717/peerj.4020 (PMC5683046; doi:10.7717/peerj.4020)
Supplement: Supplemental Information 2 [file peerj-05-4020-s002.zip › taxa_summaries.html]

Taxa Summaries


|  |  |
| --- | --- |
|  | |
| Taxonomy Summary. Current Level: | |
| View Figure (.pdf)  View Legend (.pdf) |  |
|  |


|  |
| --- |
| View Table (.txt) |

|  |  |  |  |  |  |  |
| --- | --- | --- | --- | --- | --- | --- |
|  | | Total | Isolates | P. hirsuta | P. kaalaensis | P. mollis |
| Legend | Taxonomy | % | % | % | % | % |
|  | No blast hit;Other | 0.0% | 0.0% | 0.0% | 0.0% | 0.0% |
|  | k\_\_Fungi;p\_\_Ascomycota | 96.1% | 100.0% | 98.7% | 87.9% | 97.5% |
|  | k\_\_Fungi;p\_\_Basidiomycota | 3.7% | 0.0% | 0.2% | 12.0% | 2.4% |
|  | k\_\_Fungi;p\_\_Chytridiomycota | 0.0% | 0.0% | 0.1% | 0.0% | 0.0% |
|  | k\_\_Fungi;p\_\_Zygomycota | 0.0% | 0.0% | 0.0% | 0.0% | 0.0% |
|  | k\_\_Fungi;p\_\_unidentified | 0.2% | 0.0% | 1.0% | 0.0% | 0.0% |

|  |  |
| --- | --- |
|  | |
| Taxonomy Summary. Current Level: | |
| View Figure (.pdf)  View Legend (.pdf) |  |
|  |


|  |
| --- |
| View Table (.txt) |

|  |  |  |  |  |  |  |
| --- | --- | --- | --- | --- | --- | --- |
|  | | Total | Isolates | P. hirsuta | P. kaalaensis | P. mollis |
| Legend | Taxonomy | % | % | % | % | % |
|  | No blast hit;Other;Other | 0.0% | 0.0% | 0.0% | 0.0% | 0.0% |
|  | k\_\_Fungi;p\_\_Ascomycota;c\_\_Dothideomycetes | 24.2% | 88.6% | 3.4% | 1.8% | 2.8% |
|  | k\_\_Fungi;p\_\_Ascomycota;c\_\_Eurotiomycetes | 2.0% | 3.2% | 4.6% | 0.1% | 0.1% |
|  | k\_\_Fungi;p\_\_Ascomycota;c\_\_Incertae sedis | 0.0% | 0.0% | 0.0% | 0.0% | 0.0% |
|  | k\_\_Fungi;p\_\_Ascomycota;c\_\_Lecanoromycetes | 0.0% | 0.0% | 0.0% | 0.0% | 0.0% |
|  | k\_\_Fungi;p\_\_Ascomycota;c\_\_Leotiomycetes | 58.8% | 0.0% | 80.4% | 78.2% | 76.6% |
|  | k\_\_Fungi;p\_\_Ascomycota;c\_\_Orbiliomycetes | 0.0% | 0.0% | 0.0% | 0.0% | 0.0% |
|  | k\_\_Fungi;p\_\_Ascomycota;c\_\_Saccharomycetes | 0.0% | 0.0% | 0.0% | 0.0% | 0.0% |
|  | k\_\_Fungi;p\_\_Ascomycota;c\_\_Sordariomycetes | 10.5% | 8.2% | 9.5% | 7.0% | 17.5% |
|  | k\_\_Fungi;p\_\_Ascomycota;c\_\_unidentified | 0.5% | 0.0% | 0.7% | 0.8% | 0.6% |
|  | k\_\_Fungi;p\_\_Basidiomycota;c\_\_Agaricomycetes | 0.4% | 0.0% | 0.0% | 0.6% | 0.9% |
|  | k\_\_Fungi;p\_\_Basidiomycota;c\_\_Exobasidiomycetes | 0.0% | 0.0% | 0.0% | 0.1% | 0.0% |
|  | k\_\_Fungi;p\_\_Basidiomycota;c\_\_Incertae sedis | 0.3% | 0.0% | 0.0% | 0.7% | 0.7% |
|  | k\_\_Fungi;p\_\_Basidiomycota;c\_\_Microbotryomycetes | 0.2% | 0.0% | 0.0% | 0.6% | 0.1% |
|  | k\_\_Fungi;p\_\_Basidiomycota;c\_\_Pucciniomycetes | 0.0% | 0.0% | 0.0% | 0.0% | 0.0% |
|  | k\_\_Fungi;p\_\_Basidiomycota;c\_\_Tremellomycetes | 1.0% | 0.0% | 0.1% | 3.6% | 0.1% |
|  | k\_\_Fungi;p\_\_Basidiomycota;c\_\_Ustilaginomycetes | 1.7% | 0.0% | 0.0% | 6.3% | 0.6% |
|  | k\_\_Fungi;p\_\_Chytridiomycota;c\_\_unidentified | 0.0% | 0.0% | 0.1% | 0.0% | 0.0% |
|  | k\_\_Fungi;p\_\_Zygomycota;c\_\_Incertae sedis | 0.0% | 0.0% | 0.0% | 0.0% | 0.0% |
|  | k\_\_Fungi;p\_\_unidentified;c\_\_unidentified | 0.2% | 0.0% | 1.0% | 0.0% | 0.0% |

|  |  |
| --- | --- |
|  | |
| Taxonomy Summary. Current Level: | |
| View Figure (.pdf)  View Legend (.pdf) |  |
|  |


|  |
| --- |
| View Table (.txt) |

|  |  |  |  |  |  |  |
| --- | --- | --- | --- | --- | --- | --- |
|  | | Total | Isolates | P. hirsuta | P. kaalaensis | P. mollis |
| Legend | Taxonomy | % | % | % | % | % |
|  | No blast hit;Other;Other;Other | 0.0% | 0.0% | 0.0% | 0.0% | 0.0% |
|  | k\_\_Fungi;p\_\_Ascomycota;c\_\_Dothideomycetes;o\_\_Botryosphaeriales | 0.5% | 2.0% | 0.0% | 0.0% | 0.0% |
|  | k\_\_Fungi;p\_\_Ascomycota;c\_\_Dothideomycetes;o\_\_Capnodiales | 0.4% | 0.0% | 1.6% | 0.2% | 0.0% |
|  | k\_\_Fungi;p\_\_Ascomycota;c\_\_Dothideomycetes;o\_\_Pleosporales | 23.2% | 86.6% | 1.8% | 1.6% | 2.7% |
|  | k\_\_Fungi;p\_\_Ascomycota;c\_\_Eurotiomycetes;o\_\_Chaetothyriales | 0.0% | 0.0% | 0.1% | 0.0% | 0.0% |
|  | k\_\_Fungi;p\_\_Ascomycota;c\_\_Eurotiomycetes;o\_\_Eurotiales | 2.0% | 3.2% | 4.5% | 0.1% | 0.1% |
|  | k\_\_Fungi;p\_\_Ascomycota;c\_\_Incertae sedis;o\_\_Incertae sedis | 0.0% | 0.0% | 0.0% | 0.0% | 0.0% |
|  | k\_\_Fungi;p\_\_Ascomycota;c\_\_Lecanoromycetes;o\_\_Agyriales | 0.0% | 0.0% | 0.0% | 0.0% | 0.0% |
|  | k\_\_Fungi;p\_\_Ascomycota;c\_\_Leotiomycetes;o\_\_Erysiphales | 58.5% | 0.0% | 79.3% | 78.2% | 76.5% |
|  | k\_\_Fungi;p\_\_Ascomycota;c\_\_Leotiomycetes;o\_\_Helotiales | 0.3% | 0.0% | 1.1% | 0.0% | 0.1% |
|  | k\_\_Fungi;p\_\_Ascomycota;c\_\_Leotiomycetes;o\_\_Incertae sedis | 0.0% | 0.0% | 0.0% | 0.0% | 0.0% |
|  | k\_\_Fungi;p\_\_Ascomycota;c\_\_Orbiliomycetes;o\_\_unidentified | 0.0% | 0.0% | 0.0% | 0.0% | 0.0% |
|  | k\_\_Fungi;p\_\_Ascomycota;c\_\_Saccharomycetes;o\_\_Saccharomycetales | 0.0% | 0.0% | 0.0% | 0.0% | 0.0% |
|  | k\_\_Fungi;p\_\_Ascomycota;c\_\_Sordariomycetes;o\_\_Diaporthales | 0.1% | 0.0% | 0.0% | 0.2% | 0.2% |
|  | k\_\_Fungi;p\_\_Ascomycota;c\_\_Sordariomycetes;o\_\_Hypocreales | 0.3% | 0.0% | 1.1% | 0.0% | 0.0% |
|  | k\_\_Fungi;p\_\_Ascomycota;c\_\_Sordariomycetes;o\_\_Incertae sedis | 1.3% | 4.7% | 0.3% | 0.1% | 0.0% |
|  | k\_\_Fungi;p\_\_Ascomycota;c\_\_Sordariomycetes;o\_\_Microascales | 0.1% | 0.0% | 0.5% | 0.0% | 0.0% |
|  | k\_\_Fungi;p\_\_Ascomycota;c\_\_Sordariomycetes;o\_\_Sordariales | 0.0% | 0.0% | 0.1% | 0.0% | 0.0% |
|  | k\_\_Fungi;p\_\_Ascomycota;c\_\_Sordariomycetes;o\_\_Xylariales | 2.5% | 2.8% | 5.6% | 0.9% | 0.8% |
|  | k\_\_Fungi;p\_\_Ascomycota;c\_\_Sordariomycetes;o\_\_unidentified | 6.2% | 0.6% | 2.0% | 5.8% | 16.3% |
|  | k\_\_Fungi;p\_\_Ascomycota;c\_\_unidentified;o\_\_unidentified | 0.5% | 0.0% | 0.7% | 0.8% | 0.6% |
|  | k\_\_Fungi;p\_\_Basidiomycota;c\_\_Agaricomycetes;o\_\_Agaricales | 0.0% | 0.0% | 0.0% | 0.1% | 0.0% |
|  | k\_\_Fungi;p\_\_Basidiomycota;c\_\_Agaricomycetes;o\_\_Auriculariales | 0.0% | 0.0% | 0.0% | 0.0% | 0.0% |
|  | k\_\_Fungi;p\_\_Basidiomycota;c\_\_Agaricomycetes;o\_\_Boletales | 0.1% | 0.0% | 0.0% | 0.0% | 0.5% |
|  | k\_\_Fungi;p\_\_Basidiomycota;c\_\_Agaricomycetes;o\_\_Cantharellales | 0.0% | 0.0% | 0.0% | 0.1% | 0.0% |
|  | k\_\_Fungi;p\_\_Basidiomycota;c\_\_Agaricomycetes;o\_\_Hymenochaetales | 0.1% | 0.0% | 0.0% | 0.2% | 0.2% |
|  | k\_\_Fungi;p\_\_Basidiomycota;c\_\_Agaricomycetes;o\_\_Polyporales | 0.1% | 0.0% | 0.0% | 0.2% | 0.1% |
|  | k\_\_Fungi;p\_\_Basidiomycota;c\_\_Agaricomycetes;o\_\_Russulales | 0.0% | 0.0% | 0.0% | 0.1% | 0.0% |
|  | k\_\_Fungi;p\_\_Basidiomycota;c\_\_Agaricomycetes;o\_\_unidentified | 0.0% | 0.0% | 0.0% | 0.1% | 0.1% |
|  | k\_\_Fungi;p\_\_Basidiomycota;c\_\_Exobasidiomycetes;o\_\_Incertae sedis | 0.0% | 0.0% | 0.0% | 0.1% | 0.0% |
|  | k\_\_Fungi;p\_\_Basidiomycota;c\_\_Exobasidiomycetes;o\_\_Tilletiales | 0.0% | 0.0% | 0.0% | 0.0% | 0.0% |
|  | k\_\_Fungi;p\_\_Basidiomycota;c\_\_Incertae sedis;o\_\_Malasseziales | 0.3% | 0.0% | 0.0% | 0.7% | 0.7% |
|  | k\_\_Fungi;p\_\_Basidiomycota;c\_\_Microbotryomycetes;o\_\_Sporidiobolales | 0.2% | 0.0% | 0.0% | 0.6% | 0.1% |
|  | k\_\_Fungi;p\_\_Basidiomycota;c\_\_Pucciniomycetes;o\_\_Septobasidiales | 0.0% | 0.0% | 0.0% | 0.0% | 0.0% |
|  | k\_\_Fungi;p\_\_Basidiomycota;c\_\_Tremellomycetes;o\_\_Tremellales | 1.0% | 0.0% | 0.1% | 3.6% | 0.1% |
|  | k\_\_Fungi;p\_\_Basidiomycota;c\_\_Ustilaginomycetes;o\_\_Ustilaginales | 1.7% | 0.0% | 0.0% | 6.3% | 0.6% |
|  | k\_\_Fungi;p\_\_Chytridiomycota;c\_\_unidentified;o\_\_unidentified | 0.0% | 0.0% | 0.1% | 0.0% | 0.0% |
|  | k\_\_Fungi;p\_\_Zygomycota;c\_\_Incertae sedis;o\_\_Basidiobolales | 0.0% | 0.0% | 0.0% | 0.0% | 0.0% |
|  | k\_\_Fungi;p\_\_unidentified;c\_\_unidentified;o\_\_unidentified | 0.2% | 0.0% | 1.0% | 0.0% | 0.0% |

|  |  |
| --- | --- |
|  | |
| Taxonomy Summary. Current Level: | |
| View Figure (.pdf)  View Legend (.pdf) |  |
|  |


|  |
| --- |
| View Table (.txt) |

|  |  |  |  |  |  |  |
| --- | --- | --- | --- | --- | --- | --- |
|  | | Total | Isolates | P. hirsuta | P. kaalaensis | P. mollis |
| Legend | Taxonomy | % | % | % | % | % |
|  | No blast hit;Other;Other;Other;Other | 0.0% | 0.0% | 0.0% | 0.0% | 0.0% |
|  | k\_\_Fungi;p\_\_Ascomycota;c\_\_Dothideomycetes;o\_\_Botryosphaeriales;f\_\_Botryosphaeriaceae | 0.5% | 2.0% | 0.0% | 0.0% | 0.0% |
|  | k\_\_Fungi;p\_\_Ascomycota;c\_\_Dothideomycetes;o\_\_Capnodiales;f\_\_Davidiellaceae | 0.0% | 0.0% | 0.0% | 0.0% | 0.0% |
|  | k\_\_Fungi;p\_\_Ascomycota;c\_\_Dothideomycetes;o\_\_Capnodiales;f\_\_Incertae sedis | 0.0% | 0.0% | 0.0% | 0.0% | 0.0% |
|  | k\_\_Fungi;p\_\_Ascomycota;c\_\_Dothideomycetes;o\_\_Capnodiales;f\_\_Mycosphaerellaceae | 0.4% | 0.0% | 1.6% | 0.2% | 0.0% |
|  | k\_\_Fungi;p\_\_Ascomycota;c\_\_Dothideomycetes;o\_\_Capnodiales;f\_\_Teratosphaeriaceae | 0.0% | 0.0% | 0.0% | 0.0% | 0.0% |
|  | k\_\_Fungi;p\_\_Ascomycota;c\_\_Dothideomycetes;o\_\_Pleosporales;f\_\_Cucurbitariaceae | 0.0% | 0.0% | 0.0% | 0.0% | 0.0% |
|  | k\_\_Fungi;p\_\_Ascomycota;c\_\_Dothideomycetes;o\_\_Pleosporales;f\_\_Incertae sedis | 0.1% | 0.0% | 0.4% | 0.0% | 0.0% |
|  | k\_\_Fungi;p\_\_Ascomycota;c\_\_Dothideomycetes;o\_\_Pleosporales;f\_\_Phaeosphaeriaceae | 0.2% | 0.0% | 1.0% | 0.0% | 0.0% |
|  | k\_\_Fungi;p\_\_Ascomycota;c\_\_Dothideomycetes;o\_\_Pleosporales;f\_\_Pleosporaceae | 22.1% | 86.6% | 0.0% | 0.6% | 1.1% |
|  | k\_\_Fungi;p\_\_Ascomycota;c\_\_Dothideomycetes;o\_\_Pleosporales;f\_\_Sporormiaceae | 0.0% | 0.0% | 0.0% | 0.0% | 0.0% |
|  | k\_\_Fungi;p\_\_Ascomycota;c\_\_Dothideomycetes;o\_\_Pleosporales;f\_\_unidentified | 0.8% | 0.0% | 0.4% | 1.0% | 1.6% |
|  | k\_\_Fungi;p\_\_Ascomycota;c\_\_Eurotiomycetes;o\_\_Chaetothyriales;f\_\_Herpotrichiellaceae | 0.0% | 0.0% | 0.1% | 0.0% | 0.0% |
|  | k\_\_Fungi;p\_\_Ascomycota;c\_\_Eurotiomycetes;o\_\_Chaetothyriales;f\_\_unidentified | 0.0% | 0.0% | 0.0% | 0.0% | 0.0% |
|  | k\_\_Fungi;p\_\_Ascomycota;c\_\_Eurotiomycetes;o\_\_Eurotiales;f\_\_Trichocomaceae | 2.0% | 3.2% | 4.5% | 0.1% | 0.1% |
|  | k\_\_Fungi;p\_\_Ascomycota;c\_\_Eurotiomycetes;o\_\_Eurotiales;f\_\_unidentified | 0.0% | 0.0% | 0.0% | 0.0% | 0.0% |
|  | k\_\_Fungi;p\_\_Ascomycota;c\_\_Incertae sedis;o\_\_Incertae sedis;f\_\_Incertae sedis | 0.0% | 0.0% | 0.0% | 0.0% | 0.0% |
|  | k\_\_Fungi;p\_\_Ascomycota;c\_\_Lecanoromycetes;o\_\_Agyriales;f\_\_Trapeliaceae | 0.0% | 0.0% | 0.0% | 0.0% | 0.0% |
|  | k\_\_Fungi;p\_\_Ascomycota;c\_\_Leotiomycetes;o\_\_Erysiphales;f\_\_Erysiphaceae | 58.5% | 0.0% | 79.3% | 78.2% | 76.5% |
|  | k\_\_Fungi;p\_\_Ascomycota;c\_\_Leotiomycetes;o\_\_Helotiales;f\_\_Dermateaceae | 0.0% | 0.0% | 0.0% | 0.0% | 0.0% |
|  | k\_\_Fungi;p\_\_Ascomycota;c\_\_Leotiomycetes;o\_\_Helotiales;f\_\_Hyaloscyphaceae | 0.0% | 0.0% | 0.1% | 0.0% | 0.0% |
|  | k\_\_Fungi;p\_\_Ascomycota;c\_\_Leotiomycetes;o\_\_Helotiales;f\_\_Incertae sedis | 0.1% | 0.0% | 0.5% | 0.0% | 0.1% |
|  | k\_\_Fungi;p\_\_Ascomycota;c\_\_Leotiomycetes;o\_\_Helotiales;f\_\_Sclerotiniaceae | 0.1% | 0.0% | 0.4% | 0.0% | 0.0% |
|  | k\_\_Fungi;p\_\_Ascomycota;c\_\_Leotiomycetes;o\_\_Helotiales;f\_\_unidentified | 0.0% | 0.0% | 0.0% | 0.0% | 0.0% |
|  | k\_\_Fungi;p\_\_Ascomycota;c\_\_Leotiomycetes;o\_\_Incertae sedis;f\_\_Myxotrichaceae | 0.0% | 0.0% | 0.0% | 0.0% | 0.0% |
|  | k\_\_Fungi;p\_\_Ascomycota;c\_\_Orbiliomycetes;o\_\_unidentified;f\_\_unidentified | 0.0% | 0.0% | 0.0% | 0.0% | 0.0% |
|  | k\_\_Fungi;p\_\_Ascomycota;c\_\_Saccharomycetes;o\_\_Saccharomycetales;f\_\_Dipodascaceae | 0.0% | 0.0% | 0.0% | 0.0% | 0.0% |
|  | k\_\_Fungi;p\_\_Ascomycota;c\_\_Saccharomycetes;o\_\_Saccharomycetales;f\_\_Incertae sedis | 0.0% | 0.0% | 0.0% | 0.0% | 0.0% |
|  | k\_\_Fungi;p\_\_Ascomycota;c\_\_Sordariomycetes;o\_\_Diaporthales;f\_\_Diaporthaceae | 0.1% | 0.0% | 0.0% | 0.2% | 0.2% |
|  | k\_\_Fungi;p\_\_Ascomycota;c\_\_Sordariomycetes;o\_\_Diaporthales;f\_\_Valsaceae | 0.0% | 0.0% | 0.0% | 0.0% | 0.0% |
|  | k\_\_Fungi;p\_\_Ascomycota;c\_\_Sordariomycetes;o\_\_Hypocreales;f\_\_Bionectriaceae | 0.0% | 0.0% | 0.0% | 0.0% | 0.0% |
|  | k\_\_Fungi;p\_\_Ascomycota;c\_\_Sordariomycetes;o\_\_Hypocreales;f\_\_Cordycipitaceae | 0.2% | 0.0% | 0.9% | 0.0% | 0.0% |
|  | k\_\_Fungi;p\_\_Ascomycota;c\_\_Sordariomycetes;o\_\_Hypocreales;f\_\_Hypocreaceae | 0.0% | 0.0% | 0.1% | 0.0% | 0.0% |
|  | k\_\_Fungi;p\_\_Ascomycota;c\_\_Sordariomycetes;o\_\_Hypocreales;f\_\_Incertae sedis | 0.0% | 0.0% | 0.1% | 0.0% | 0.0% |
|  | k\_\_Fungi;p\_\_Ascomycota;c\_\_Sordariomycetes;o\_\_Hypocreales;f\_\_Nectriaceae | 0.0% | 0.0% | 0.0% | 0.0% | 0.0% |
|  | k\_\_Fungi;p\_\_Ascomycota;c\_\_Sordariomycetes;o\_\_Hypocreales;f\_\_unidentified | 0.0% | 0.0% | 0.0% | 0.0% | 0.0% |
|  | k\_\_Fungi;p\_\_Ascomycota;c\_\_Sordariomycetes;o\_\_Incertae sedis;f\_\_Glomerellaceae | 1.3% | 4.7% | 0.3% | 0.1% | 0.0% |
|  | k\_\_Fungi;p\_\_Ascomycota;c\_\_Sordariomycetes;o\_\_Microascales;f\_\_Ceratocystidaceae | 0.0% | 0.0% | 0.0% | 0.0% | 0.0% |
|  | k\_\_Fungi;p\_\_Ascomycota;c\_\_Sordariomycetes;o\_\_Microascales;f\_\_Halosphaeriaceae | 0.0% | 0.0% | 0.0% | 0.0% | 0.0% |
|  | k\_\_Fungi;p\_\_Ascomycota;c\_\_Sordariomycetes;o\_\_Microascales;f\_\_Microascaceae | 0.1% | 0.0% | 0.5% | 0.0% | 0.0% |
|  | k\_\_Fungi;p\_\_Ascomycota;c\_\_Sordariomycetes;o\_\_Sordariales;f\_\_Chaetomiaceae | 0.0% | 0.0% | 0.0% | 0.0% | 0.0% |
|  | k\_\_Fungi;p\_\_Ascomycota;c\_\_Sordariomycetes;o\_\_Sordariales;f\_\_Lasiosphaeriaceae | 0.0% | 0.0% | 0.0% | 0.0% | 0.0% |
|  | k\_\_Fungi;p\_\_Ascomycota;c\_\_Sordariomycetes;o\_\_Xylariales;f\_\_Amphisphaeriaceae | 1.4% | 0.0% | 5.4% | 0.1% | 0.1% |
|  | k\_\_Fungi;p\_\_Ascomycota;c\_\_Sordariomycetes;o\_\_Xylariales;f\_\_Xylariaceae | 1.1% | 2.8% | 0.1% | 0.9% | 0.7% |
|  | k\_\_Fungi;p\_\_Ascomycota;c\_\_Sordariomycetes;o\_\_Xylariales;f\_\_unidentified | 0.0% | 0.0% | 0.0% | 0.0% | 0.0% |
|  | k\_\_Fungi;p\_\_Ascomycota;c\_\_Sordariomycetes;o\_\_unidentified;f\_\_unidentified | 6.2% | 0.6% | 2.0% | 5.8% | 16.3% |
|  | k\_\_Fungi;p\_\_Ascomycota;c\_\_unidentified;o\_\_unidentified;f\_\_unidentified | 0.5% | 0.0% | 0.7% | 0.8% | 0.6% |
|  | k\_\_Fungi;p\_\_Basidiomycota;c\_\_Agaricomycetes;o\_\_Agaricales;f\_\_Entolomataceae | 0.0% | 0.0% | 0.0% | 0.0% | 0.0% |
|  | k\_\_Fungi;p\_\_Basidiomycota;c\_\_Agaricomycetes;o\_\_Agaricales;f\_\_Marasmiaceae | 0.0% | 0.0% | 0.0% | 0.1% | 0.0% |
|  | k\_\_Fungi;p\_\_Basidiomycota;c\_\_Agaricomycetes;o\_\_Agaricales;f\_\_Schizophyllaceae | 0.0% | 0.0% | 0.0% | 0.0% | 0.0% |
|  | k\_\_Fungi;p\_\_Basidiomycota;c\_\_Agaricomycetes;o\_\_Auriculariales;f\_\_Incertae sedis | 0.0% | 0.0% | 0.0% | 0.0% | 0.0% |
|  | k\_\_Fungi;p\_\_Basidiomycota;c\_\_Agaricomycetes;o\_\_Boletales;f\_\_Coniophoraceae | 0.0% | 0.0% | 0.0% | 0.0% | 0.0% |
|  | k\_\_Fungi;p\_\_Basidiomycota;c\_\_Agaricomycetes;o\_\_Boletales;f\_\_Sclerodermataceae | 0.1% | 0.0% | 0.0% | 0.0% | 0.5% |
|  | k\_\_Fungi;p\_\_Basidiomycota;c\_\_Agaricomycetes;o\_\_Cantharellales;f\_\_Ceratobasidiaceae | 0.0% | 0.0% | 0.0% | 0.1% | 0.0% |
|  | k\_\_Fungi;p\_\_Basidiomycota;c\_\_Agaricomycetes;o\_\_Hymenochaetales;f\_\_Hymenochaetaceae | 0.1% | 0.0% | 0.0% | 0.2% | 0.2% |
|  | k\_\_Fungi;p\_\_Basidiomycota;c\_\_Agaricomycetes;o\_\_Polyporales;f\_\_Phanerochaetaceae | 0.0% | 0.0% | 0.0% | 0.0% | 0.0% |
|  | k\_\_Fungi;p\_\_Basidiomycota;c\_\_Agaricomycetes;o\_\_Polyporales;f\_\_Polyporaceae | 0.0% | 0.0% | 0.0% | 0.1% | 0.0% |
|  | k\_\_Fungi;p\_\_Basidiomycota;c\_\_Agaricomycetes;o\_\_Polyporales;f\_\_unidentified | 0.0% | 0.0% | 0.0% | 0.0% | 0.1% |
|  | k\_\_Fungi;p\_\_Basidiomycota;c\_\_Agaricomycetes;o\_\_Russulales;f\_\_Bondarzewiaceae | 0.0% | 0.0% | 0.0% | 0.0% | 0.0% |
|  | k\_\_Fungi;p\_\_Basidiomycota;c\_\_Agaricomycetes;o\_\_Russulales;f\_\_Peniophoraceae | 0.0% | 0.0% | 0.0% | 0.0% | 0.0% |
|  | k\_\_Fungi;p\_\_Basidiomycota;c\_\_Agaricomycetes;o\_\_unidentified;f\_\_unidentified | 0.0% | 0.0% | 0.0% | 0.1% | 0.1% |
|  | k\_\_Fungi;p\_\_Basidiomycota;c\_\_Exobasidiomycetes;o\_\_Incertae sedis;f\_\_Incertae sedis | 0.0% | 0.0% | 0.0% | 0.1% | 0.0% |
|  | k\_\_Fungi;p\_\_Basidiomycota;c\_\_Exobasidiomycetes;o\_\_Tilletiales;f\_\_Tilletiaceae | 0.0% | 0.0% | 0.0% | 0.0% | 0.0% |
|  | k\_\_Fungi;p\_\_Basidiomycota;c\_\_Incertae sedis;o\_\_Malasseziales;f\_\_Malasseziaceae | 0.3% | 0.0% | 0.0% | 0.6% | 0.5% |
|  | k\_\_Fungi;p\_\_Basidiomycota;c\_\_Incertae sedis;o\_\_Malasseziales;f\_\_unidentified | 0.1% | 0.0% | 0.0% | 0.2% | 0.1% |
|  | k\_\_Fungi;p\_\_Basidiomycota;c\_\_Microbotryomycetes;o\_\_Sporidiobolales;f\_\_Incertae sedis | 0.2% | 0.0% | 0.0% | 0.6% | 0.1% |
|  | k\_\_Fungi;p\_\_Basidiomycota;c\_\_Microbotryomycetes;o\_\_Sporidiobolales;f\_\_unidentified | 0.0% | 0.0% | 0.0% | 0.0% | 0.0% |
|  | k\_\_Fungi;p\_\_Basidiomycota;c\_\_Pucciniomycetes;o\_\_Septobasidiales;f\_\_Septobasidiaceae | 0.0% | 0.0% | 0.0% | 0.0% | 0.0% |
|  | k\_\_Fungi;p\_\_Basidiomycota;c\_\_Tremellomycetes;o\_\_Tremellales;f\_\_Incertae sedis | 1.0% | 0.0% | 0.1% | 3.6% | 0.1% |
|  | k\_\_Fungi;p\_\_Basidiomycota;c\_\_Ustilaginomycetes;o\_\_Ustilaginales;f\_\_Ustilaginaceae | 1.7% | 0.0% | 0.0% | 6.3% | 0.6% |
|  | k\_\_Fungi;p\_\_Chytridiomycota;c\_\_unidentified;o\_\_unidentified;f\_\_unidentified | 0.0% | 0.0% | 0.1% | 0.0% | 0.0% |
|  | k\_\_Fungi;p\_\_Zygomycota;c\_\_Incertae sedis;o\_\_Basidiobolales;f\_\_Basidiobolaceae | 0.0% | 0.0% | 0.0% | 0.0% | 0.0% |
|  | k\_\_Fungi;p\_\_unidentified;c\_\_unidentified;o\_\_unidentified;f\_\_unidentified | 0.2% | 0.0% | 1.0% | 0.0% | 0.0% |

|  |  |
| --- | --- |
|  | |
| Taxonomy Summary. Current Level: | |
| View Figure (.pdf)  View Legend (.pdf) |  |
|  |


|  |
| --- |
| View Table (.txt) |

|  |  |  |  |  |  |  |
| --- | --- | --- | --- | --- | --- | --- |
|  | | Total | Isolates | P. hirsuta | P. kaalaensis | P. mollis |
| Legend | Taxonomy | % | % | % | % | % |
|  | No blast hit;Other;Other;Other;Other;Other | 0.0% | 0.0% | 0.0% | 0.0% | 0.0% |
|  | k\_\_Fungi;p\_\_Ascomycota;c\_\_Dothideomycetes;o\_\_Botryosphaeriales;f\_\_Botryosphaeriaceae;g\_\_Neofusicoccum | 0.3% | 1.3% | 0.0% | 0.0% | 0.0% |
|  | k\_\_Fungi;p\_\_Ascomycota;c\_\_Dothideomycetes;o\_\_Botryosphaeriales;f\_\_Botryosphaeriaceae;g\_\_Phyllosticta | 0.2% | 0.8% | 0.0% | 0.0% | 0.0% |
|  | k\_\_Fungi;p\_\_Ascomycota;c\_\_Dothideomycetes;o\_\_Capnodiales;f\_\_Davidiellaceae;g\_\_Cladosporium | 0.0% | 0.0% | 0.0% | 0.0% | 0.0% |
|  | k\_\_Fungi;p\_\_Ascomycota;c\_\_Dothideomycetes;o\_\_Capnodiales;f\_\_Incertae sedis;g\_\_Capnobotryella | 0.0% | 0.0% | 0.0% | 0.0% | 0.0% |
|  | k\_\_Fungi;p\_\_Ascomycota;c\_\_Dothideomycetes;o\_\_Capnodiales;f\_\_Mycosphaerellaceae;g\_\_Mycosphaerella | 0.0% | 0.0% | 0.1% | 0.0% | 0.0% |
|  | k\_\_Fungi;p\_\_Ascomycota;c\_\_Dothideomycetes;o\_\_Capnodiales;f\_\_Mycosphaerellaceae;g\_\_Passalora | 0.0% | 0.0% | 0.0% | 0.0% | 0.0% |
|  | k\_\_Fungi;p\_\_Ascomycota;c\_\_Dothideomycetes;o\_\_Capnodiales;f\_\_Mycosphaerellaceae;g\_\_Phaeophleospora | 0.3% | 0.0% | 1.4% | 0.0% | 0.0% |
|  | k\_\_Fungi;p\_\_Ascomycota;c\_\_Dothideomycetes;o\_\_Capnodiales;f\_\_Mycosphaerellaceae;g\_\_Pseudocercospora | 0.1% | 0.0% | 0.1% | 0.2% | 0.0% |
|  | k\_\_Fungi;p\_\_Ascomycota;c\_\_Dothideomycetes;o\_\_Capnodiales;f\_\_Mycosphaerellaceae;g\_\_Stenella | 0.0% | 0.0% | 0.0% | 0.0% | 0.0% |
|  | k\_\_Fungi;p\_\_Ascomycota;c\_\_Dothideomycetes;o\_\_Capnodiales;f\_\_Teratosphaeriaceae;g\_\_Devriesia | 0.0% | 0.0% | 0.0% | 0.0% | 0.0% |
|  | k\_\_Fungi;p\_\_Ascomycota;c\_\_Dothideomycetes;o\_\_Pleosporales;f\_\_Cucurbitariaceae;g\_\_Pyrenochaetopsis | 0.0% | 0.0% | 0.0% | 0.0% | 0.0% |
|  | k\_\_Fungi;p\_\_Ascomycota;c\_\_Dothideomycetes;o\_\_Pleosporales;f\_\_Incertae sedis;g\_\_Phoma | 0.1% | 0.0% | 0.4% | 0.0% | 0.0% |
|  | k\_\_Fungi;p\_\_Ascomycota;c\_\_Dothideomycetes;o\_\_Pleosporales;f\_\_Phaeosphaeriaceae;g\_\_Ampelomyces | 0.2% | 0.0% | 1.0% | 0.0% | 0.0% |
|  | k\_\_Fungi;p\_\_Ascomycota;c\_\_Dothideomycetes;o\_\_Pleosporales;f\_\_Phaeosphaeriaceae;g\_\_Phaeosphaeria | 0.0% | 0.0% | 0.0% | 0.0% | 0.0% |
|  | k\_\_Fungi;p\_\_Ascomycota;c\_\_Dothideomycetes;o\_\_Pleosporales;f\_\_Pleosporaceae;g\_\_Alternaria | 21.9% | 86.6% | 0.0% | 0.0% | 0.8% |
|  | k\_\_Fungi;p\_\_Ascomycota;c\_\_Dothideomycetes;o\_\_Pleosporales;f\_\_Pleosporaceae;g\_\_Curvularia | 0.0% | 0.0% | 0.0% | 0.1% | 0.0% |
|  | k\_\_Fungi;p\_\_Ascomycota;c\_\_Dothideomycetes;o\_\_Pleosporales;f\_\_Pleosporaceae;g\_\_Epicoccum | 0.1% | 0.0% | 0.0% | 0.2% | 0.1% |
|  | k\_\_Fungi;p\_\_Ascomycota;c\_\_Dothideomycetes;o\_\_Pleosporales;f\_\_Pleosporaceae;g\_\_unidentified | 0.1% | 0.0% | 0.0% | 0.2% | 0.2% |
|  | k\_\_Fungi;p\_\_Ascomycota;c\_\_Dothideomycetes;o\_\_Pleosporales;f\_\_Sporormiaceae;g\_\_unidentified | 0.0% | 0.0% | 0.0% | 0.0% | 0.0% |
|  | k\_\_Fungi;p\_\_Ascomycota;c\_\_Dothideomycetes;o\_\_Pleosporales;f\_\_unidentified;g\_\_unidentified | 0.8% | 0.0% | 0.4% | 1.0% | 1.6% |
|  | k\_\_Fungi;p\_\_Ascomycota;c\_\_Eurotiomycetes;o\_\_Chaetothyriales;f\_\_Herpotrichiellaceae;g\_\_Cladophialophora | 0.0% | 0.0% | 0.0% | 0.0% | 0.0% |
|  | k\_\_Fungi;p\_\_Ascomycota;c\_\_Eurotiomycetes;o\_\_Chaetothyriales;f\_\_Herpotrichiellaceae;g\_\_Exophiala | 0.0% | 0.0% | 0.1% | 0.0% | 0.0% |
|  | k\_\_Fungi;p\_\_Ascomycota;c\_\_Eurotiomycetes;o\_\_Chaetothyriales;f\_\_unidentified;g\_\_unidentified | 0.0% | 0.0% | 0.0% | 0.0% | 0.0% |
|  | k\_\_Fungi;p\_\_Ascomycota;c\_\_Eurotiomycetes;o\_\_Eurotiales;f\_\_Trichocomaceae;g\_\_Aspergillus | 1.2% | 0.0% | 4.5% | 0.1% | 0.0% |
|  | k\_\_Fungi;p\_\_Ascomycota;c\_\_Eurotiomycetes;o\_\_Eurotiales;f\_\_Trichocomaceae;g\_\_Penicillium | 0.8% | 3.2% | 0.0% | 0.0% | 0.0% |
|  | k\_\_Fungi;p\_\_Ascomycota;c\_\_Eurotiomycetes;o\_\_Eurotiales;f\_\_unidentified;g\_\_unidentified | 0.0% | 0.0% | 0.0% | 0.0% | 0.0% |
|  | k\_\_Fungi;p\_\_Ascomycota;c\_\_Incertae sedis;o\_\_Incertae sedis;f\_\_Incertae sedis;g\_\_Alpakesa | 0.0% | 0.0% | 0.0% | 0.0% | 0.0% |
|  | k\_\_Fungi;p\_\_Ascomycota;c\_\_Incertae sedis;o\_\_Incertae sedis;f\_\_Incertae sedis;g\_\_Canalisporium | 0.0% | 0.0% | 0.0% | 0.0% | 0.0% |
|  | k\_\_Fungi;p\_\_Ascomycota;c\_\_Lecanoromycetes;o\_\_Agyriales;f\_\_Trapeliaceae;g\_\_Sarea | 0.0% | 0.0% | 0.0% | 0.0% | 0.0% |
|  | k\_\_Fungi;p\_\_Ascomycota;c\_\_Leotiomycetes;o\_\_Erysiphales;f\_\_Erysiphaceae;g\_\_Neoerysiphe | 58.5% | 0.0% | 79.3% | 78.2% | 76.5% |
|  | k\_\_Fungi;p\_\_Ascomycota;c\_\_Leotiomycetes;o\_\_Helotiales;f\_\_Dermateaceae;g\_\_unidentified | 0.0% | 0.0% | 0.0% | 0.0% | 0.0% |
|  | k\_\_Fungi;p\_\_Ascomycota;c\_\_Leotiomycetes;o\_\_Helotiales;f\_\_Hyaloscyphaceae;g\_\_Microscypha | 0.0% | 0.0% | 0.1% | 0.0% | 0.0% |
|  | k\_\_Fungi;p\_\_Ascomycota;c\_\_Leotiomycetes;o\_\_Helotiales;f\_\_Incertae sedis;g\_\_Cadophora | 0.0% | 0.0% | 0.0% | 0.0% | 0.0% |
|  | k\_\_Fungi;p\_\_Ascomycota;c\_\_Leotiomycetes;o\_\_Helotiales;f\_\_Incertae sedis;g\_\_Chlorencoelia | 0.0% | 0.0% | 0.1% | 0.0% | 0.0% |
|  | k\_\_Fungi;p\_\_Ascomycota;c\_\_Leotiomycetes;o\_\_Helotiales;f\_\_Incertae sedis;g\_\_Scleropezicula | 0.0% | 0.0% | 0.0% | 0.0% | 0.1% |
|  | k\_\_Fungi;p\_\_Ascomycota;c\_\_Leotiomycetes;o\_\_Helotiales;f\_\_Incertae sedis;g\_\_Tetracladium | 0.1% | 0.0% | 0.3% | 0.0% | 0.0% |
|  | k\_\_Fungi;p\_\_Ascomycota;c\_\_Leotiomycetes;o\_\_Helotiales;f\_\_Sclerotiniaceae;g\_\_Botrytis | 0.1% | 0.0% | 0.4% | 0.0% | 0.0% |
|  | k\_\_Fungi;p\_\_Ascomycota;c\_\_Leotiomycetes;o\_\_Helotiales;f\_\_unidentified;g\_\_unidentified | 0.0% | 0.0% | 0.0% | 0.0% | 0.0% |
|  | k\_\_Fungi;p\_\_Ascomycota;c\_\_Leotiomycetes;o\_\_Incertae sedis;f\_\_Myxotrichaceae;g\_\_Oidiodendron | 0.0% | 0.0% | 0.0% | 0.0% | 0.0% |
|  | k\_\_Fungi;p\_\_Ascomycota;c\_\_Orbiliomycetes;o\_\_unidentified;f\_\_unidentified;g\_\_unidentified | 0.0% | 0.0% | 0.0% | 0.0% | 0.0% |
|  | k\_\_Fungi;p\_\_Ascomycota;c\_\_Saccharomycetes;o\_\_Saccharomycetales;f\_\_Dipodascaceae;g\_\_Dipodascus | 0.0% | 0.0% | 0.0% | 0.0% | 0.0% |
|  | k\_\_Fungi;p\_\_Ascomycota;c\_\_Saccharomycetes;o\_\_Saccharomycetales;f\_\_Incertae sedis;g\_\_Debaryomyces | 0.0% | 0.0% | 0.0% | 0.0% | 0.0% |
|  | k\_\_Fungi;p\_\_Ascomycota;c\_\_Sordariomycetes;o\_\_Diaporthales;f\_\_Diaporthaceae;g\_\_Diaporthe | 0.1% | 0.0% | 0.0% | 0.2% | 0.2% |
|  | k\_\_Fungi;p\_\_Ascomycota;c\_\_Sordariomycetes;o\_\_Diaporthales;f\_\_Valsaceae;g\_\_Cytospora | 0.0% | 0.0% | 0.0% | 0.0% | 0.0% |
|  | k\_\_Fungi;p\_\_Ascomycota;c\_\_Sordariomycetes;o\_\_Hypocreales;f\_\_Bionectriaceae;g\_\_Hydropisphaera | 0.0% | 0.0% | 0.0% | 0.0% | 0.0% |
|  | k\_\_Fungi;p\_\_Ascomycota;c\_\_Sordariomycetes;o\_\_Hypocreales;f\_\_Cordycipitaceae;g\_\_Beauveria | 0.2% | 0.0% | 0.9% | 0.0% | 0.0% |
|  | k\_\_Fungi;p\_\_Ascomycota;c\_\_Sordariomycetes;o\_\_Hypocreales;f\_\_Cordycipitaceae;g\_\_Lecanicillium | 0.0% | 0.0% | 0.0% | 0.0% | 0.0% |
|  | k\_\_Fungi;p\_\_Ascomycota;c\_\_Sordariomycetes;o\_\_Hypocreales;f\_\_Cordycipitaceae;g\_\_unidentified | 0.0% | 0.0% | 0.0% | 0.0% | 0.0% |
|  | k\_\_Fungi;p\_\_Ascomycota;c\_\_Sordariomycetes;o\_\_Hypocreales;f\_\_Hypocreaceae;g\_\_Sepedonium | 0.0% | 0.0% | 0.1% | 0.0% | 0.0% |
|  | k\_\_Fungi;p\_\_Ascomycota;c\_\_Sordariomycetes;o\_\_Hypocreales;f\_\_Incertae sedis;g\_\_Myrothecium | 0.0% | 0.0% | 0.0% | 0.0% | 0.0% |
|  | k\_\_Fungi;p\_\_Ascomycota;c\_\_Sordariomycetes;o\_\_Hypocreales;f\_\_Incertae sedis;g\_\_Stachybotrys | 0.0% | 0.0% | 0.1% | 0.0% | 0.0% |
|  | k\_\_Fungi;p\_\_Ascomycota;c\_\_Sordariomycetes;o\_\_Hypocreales;f\_\_Nectriaceae;g\_\_Fusarium | 0.0% | 0.0% | 0.0% | 0.0% | 0.0% |
|  | k\_\_Fungi;p\_\_Ascomycota;c\_\_Sordariomycetes;o\_\_Hypocreales;f\_\_Nectriaceae;g\_\_unidentified | 0.0% | 0.0% | 0.0% | 0.0% | 0.0% |
|  | k\_\_Fungi;p\_\_Ascomycota;c\_\_Sordariomycetes;o\_\_Hypocreales;f\_\_unidentified;g\_\_unidentified | 0.0% | 0.0% | 0.0% | 0.0% | 0.0% |
|  | k\_\_Fungi;p\_\_Ascomycota;c\_\_Sordariomycetes;o\_\_Incertae sedis;f\_\_Glomerellaceae;g\_\_Colletotrichum | 1.3% | 4.7% | 0.3% | 0.1% | 0.0% |
|  | k\_\_Fungi;p\_\_Ascomycota;c\_\_Sordariomycetes;o\_\_Microascales;f\_\_Ceratocystidaceae;g\_\_Ambrosiella | 0.0% | 0.0% | 0.0% | 0.0% | 0.0% |
|  | k\_\_Fungi;p\_\_Ascomycota;c\_\_Sordariomycetes;o\_\_Microascales;f\_\_Halosphaeriaceae;g\_\_unidentified | 0.0% | 0.0% | 0.0% | 0.0% | 0.0% |
|  | k\_\_Fungi;p\_\_Ascomycota;c\_\_Sordariomycetes;o\_\_Microascales;f\_\_Microascaceae;g\_\_Graphium | 0.1% | 0.0% | 0.5% | 0.0% | 0.0% |
|  | k\_\_Fungi;p\_\_Ascomycota;c\_\_Sordariomycetes;o\_\_Sordariales;f\_\_Chaetomiaceae;g\_\_Chaetomium | 0.0% | 0.0% | 0.0% | 0.0% | 0.0% |
|  | k\_\_Fungi;p\_\_Ascomycota;c\_\_Sordariomycetes;o\_\_Sordariales;f\_\_Lasiosphaeriaceae;g\_\_unidentified | 0.0% | 0.0% | 0.0% | 0.0% | 0.0% |
|  | k\_\_Fungi;p\_\_Ascomycota;c\_\_Sordariomycetes;o\_\_Xylariales;f\_\_Amphisphaeriaceae;g\_\_Neopestalotiopsis | 1.4% | 0.0% | 5.3% | 0.1% | 0.1% |
|  | k\_\_Fungi;p\_\_Ascomycota;c\_\_Sordariomycetes;o\_\_Xylariales;f\_\_Amphisphaeriaceae;g\_\_Pestalotiopsis | 0.0% | 0.0% | 0.1% | 0.0% | 0.0% |
|  | k\_\_Fungi;p\_\_Ascomycota;c\_\_Sordariomycetes;o\_\_Xylariales;f\_\_Amphisphaeriaceae;g\_\_Seiridium | 0.0% | 0.0% | 0.0% | 0.0% | 0.0% |
|  | k\_\_Fungi;p\_\_Ascomycota;c\_\_Sordariomycetes;o\_\_Xylariales;f\_\_Xylariaceae;g\_\_Annulohypoxylon | 0.0% | 0.0% | 0.0% | 0.0% | 0.0% |
|  | k\_\_Fungi;p\_\_Ascomycota;c\_\_Sordariomycetes;o\_\_Xylariales;f\_\_Xylariaceae;g\_\_Biscogniauxia | 0.0% | 0.0% | 0.0% | 0.0% | 0.0% |
|  | k\_\_Fungi;p\_\_Ascomycota;c\_\_Sordariomycetes;o\_\_Xylariales;f\_\_Xylariaceae;g\_\_Hypoxylon | 0.4% | 0.0% | 0.0% | 0.8% | 0.6% |
|  | k\_\_Fungi;p\_\_Ascomycota;c\_\_Sordariomycetes;o\_\_Xylariales;f\_\_Xylariaceae;g\_\_Kretzschmaria | 0.0% | 0.0% | 0.0% | 0.0% | 0.0% |
|  | k\_\_Fungi;p\_\_Ascomycota;c\_\_Sordariomycetes;o\_\_Xylariales;f\_\_Xylariaceae;g\_\_Muscodor | 0.0% | 0.0% | 0.0% | 0.0% | 0.0% |
|  | k\_\_Fungi;p\_\_Ascomycota;c\_\_Sordariomycetes;o\_\_Xylariales;f\_\_Xylariaceae;g\_\_Xylaria | 0.0% | 0.0% | 0.0% | 0.0% | 0.1% |
|  | k\_\_Fungi;p\_\_Ascomycota;c\_\_Sordariomycetes;o\_\_Xylariales;f\_\_Xylariaceae;g\_\_unidentified | 0.7% | 2.8% | 0.1% | 0.1% | 0.0% |
|  | k\_\_Fungi;p\_\_Ascomycota;c\_\_Sordariomycetes;o\_\_Xylariales;f\_\_unidentified;g\_\_unidentified | 0.0% | 0.0% | 0.0% | 0.0% | 0.0% |
|  | k\_\_Fungi;p\_\_Ascomycota;c\_\_Sordariomycetes;o\_\_unidentified;f\_\_unidentified;g\_\_unidentified | 6.2% | 0.6% | 2.0% | 5.8% | 16.3% |
|  | k\_\_Fungi;p\_\_Ascomycota;c\_\_unidentified;o\_\_unidentified;f\_\_unidentified;g\_\_unidentified | 0.5% | 0.0% | 0.7% | 0.8% | 0.6% |
|  | k\_\_Fungi;p\_\_Basidiomycota;c\_\_Agaricomycetes;o\_\_Agaricales;f\_\_Entolomataceae;g\_\_unidentified | 0.0% | 0.0% | 0.0% | 0.0% | 0.0% |
|  | k\_\_Fungi;p\_\_Basidiomycota;c\_\_Agaricomycetes;o\_\_Agaricales;f\_\_Marasmiaceae;g\_\_unidentified | 0.0% | 0.0% | 0.0% | 0.1% | 0.0% |
|  | k\_\_Fungi;p\_\_Basidiomycota;c\_\_Agaricomycetes;o\_\_Agaricales;f\_\_Schizophyllaceae;g\_\_Schizophyllum | 0.0% | 0.0% | 0.0% | 0.0% | 0.0% |
|  | k\_\_Fungi;p\_\_Basidiomycota;c\_\_Agaricomycetes;o\_\_Auriculariales;f\_\_Incertae sedis;g\_\_Auricularia | 0.0% | 0.0% | 0.0% | 0.0% | 0.0% |
|  | k\_\_Fungi;p\_\_Basidiomycota;c\_\_Agaricomycetes;o\_\_Boletales;f\_\_Coniophoraceae;g\_\_Coniophora | 0.0% | 0.0% | 0.0% | 0.0% | 0.0% |
|  | k\_\_Fungi;p\_\_Basidiomycota;c\_\_Agaricomycetes;o\_\_Boletales;f\_\_Sclerodermataceae;g\_\_Scleroderma | 0.1% | 0.0% | 0.0% | 0.0% | 0.5% |
|  | k\_\_Fungi;p\_\_Basidiomycota;c\_\_Agaricomycetes;o\_\_Cantharellales;f\_\_Ceratobasidiaceae;g\_\_unidentified | 0.0% | 0.0% | 0.0% | 0.1% | 0.0% |
|  | k\_\_Fungi;p\_\_Basidiomycota;c\_\_Agaricomycetes;o\_\_Hymenochaetales;f\_\_Hymenochaetaceae;g\_\_Phellinus | 0.1% | 0.0% | 0.0% | 0.2% | 0.2% |
|  | k\_\_Fungi;p\_\_Basidiomycota;c\_\_Agaricomycetes;o\_\_Polyporales;f\_\_Phanerochaetaceae;g\_\_Ceriporia | 0.0% | 0.0% | 0.0% | 0.0% | 0.0% |
|  | k\_\_Fungi;p\_\_Basidiomycota;c\_\_Agaricomycetes;o\_\_Polyporales;f\_\_Polyporaceae;g\_\_Funalia | 0.0% | 0.0% | 0.0% | 0.1% | 0.0% |
|  | k\_\_Fungi;p\_\_Basidiomycota;c\_\_Agaricomycetes;o\_\_Polyporales;f\_\_unidentified;g\_\_unidentified | 0.0% | 0.0% | 0.0% | 0.0% | 0.1% |
|  | k\_\_Fungi;p\_\_Basidiomycota;c\_\_Agaricomycetes;o\_\_Russulales;f\_\_Bondarzewiaceae;g\_\_Gloiodon | 0.0% | 0.0% | 0.0% | 0.0% | 0.0% |
|  | k\_\_Fungi;p\_\_Basidiomycota;c\_\_Agaricomycetes;o\_\_Russulales;f\_\_Bondarzewiaceae;g\_\_Heterobasidion | 0.0% | 0.0% | 0.0% | 0.0% | 0.0% |
|  | k\_\_Fungi;p\_\_Basidiomycota;c\_\_Agaricomycetes;o\_\_Russulales;f\_\_Peniophoraceae;g\_\_Peniophora | 0.0% | 0.0% | 0.0% | 0.0% | 0.0% |
|  | k\_\_Fungi;p\_\_Basidiomycota;c\_\_Agaricomycetes;o\_\_unidentified;f\_\_unidentified;g\_\_unidentified | 0.0% | 0.0% | 0.0% | 0.1% | 0.1% |
|  | k\_\_Fungi;p\_\_Basidiomycota;c\_\_Exobasidiomycetes;o\_\_Incertae sedis;f\_\_Incertae sedis;g\_\_Tilletiopsis | 0.0% | 0.0% | 0.0% | 0.1% | 0.0% |
|  | k\_\_Fungi;p\_\_Basidiomycota;c\_\_Exobasidiomycetes;o\_\_Tilletiales;f\_\_Tilletiaceae;g\_\_Tilletia | 0.0% | 0.0% | 0.0% | 0.0% | 0.0% |
|  | k\_\_Fungi;p\_\_Basidiomycota;c\_\_Incertae sedis;o\_\_Malasseziales;f\_\_Malasseziaceae;g\_\_Malassezia | 0.3% | 0.0% | 0.0% | 0.6% | 0.5% |
|  | k\_\_Fungi;p\_\_Basidiomycota;c\_\_Incertae sedis;o\_\_Malasseziales;f\_\_unidentified;g\_\_unidentified | 0.1% | 0.0% | 0.0% | 0.2% | 0.1% |
|  | k\_\_Fungi;p\_\_Basidiomycota;c\_\_Microbotryomycetes;o\_\_Sporidiobolales;f\_\_Incertae sedis;g\_\_Rhodotorula | 0.2% | 0.0% | 0.0% | 0.6% | 0.1% |
|  | k\_\_Fungi;p\_\_Basidiomycota;c\_\_Microbotryomycetes;o\_\_Sporidiobolales;f\_\_Incertae sedis;g\_\_Sporobolomyces | 0.0% | 0.0% | 0.0% | 0.0% | 0.0% |
|  | k\_\_Fungi;p\_\_Basidiomycota;c\_\_Microbotryomycetes;o\_\_Sporidiobolales;f\_\_unidentified;g\_\_unidentified | 0.0% | 0.0% | 0.0% | 0.0% | 0.0% |
|  | k\_\_Fungi;p\_\_Basidiomycota;c\_\_Pucciniomycetes;o\_\_Septobasidiales;f\_\_Septobasidiaceae;g\_\_Septobasidium | 0.0% | 0.0% | 0.0% | 0.0% | 0.0% |
|  | k\_\_Fungi;p\_\_Basidiomycota;c\_\_Tremellomycetes;o\_\_Tremellales;f\_\_Incertae sedis;g\_\_Cryptococcus | 0.9% | 0.0% | 0.1% | 3.6% | 0.1% |
|  | k\_\_Fungi;p\_\_Basidiomycota;c\_\_Tremellomycetes;o\_\_Tremellales;f\_\_Incertae sedis;g\_\_Derxomyces | 0.0% | 0.0% | 0.0% | 0.0% | 0.0% |
|  | k\_\_Fungi;p\_\_Basidiomycota;c\_\_Tremellomycetes;o\_\_Tremellales;f\_\_Incertae sedis;g\_\_Hannaella | 0.0% | 0.0% | 0.0% | 0.0% | 0.0% |
|  | k\_\_Fungi;p\_\_Basidiomycota;c\_\_Tremellomycetes;o\_\_Tremellales;f\_\_Incertae sedis;g\_\_Tremella | 0.0% | 0.0% | 0.0% | 0.0% | 0.0% |
|  | k\_\_Fungi;p\_\_Basidiomycota;c\_\_Tremellomycetes;o\_\_Tremellales;f\_\_Incertae sedis;g\_\_unidentified | 0.0% | 0.0% | 0.0% | 0.0% | 0.0% |
|  | k\_\_Fungi;p\_\_Basidiomycota;c\_\_Ustilaginomycetes;o\_\_Ustilaginales;f\_\_Ustilaginaceae;g\_\_Pseudozyma | 1.7% | 0.0% | 0.0% | 6.3% | 0.6% |
|  | k\_\_Fungi;p\_\_Chytridiomycota;c\_\_unidentified;o\_\_unidentified;f\_\_unidentified;g\_\_unidentified | 0.0% | 0.0% | 0.1% | 0.0% | 0.0% |
|  | k\_\_Fungi;p\_\_Zygomycota;c\_\_Incertae sedis;o\_\_Basidiobolales;f\_\_Basidiobolaceae;g\_\_Basidiobolus | 0.0% | 0.0% | 0.0% | 0.0% | 0.0% |
|  | k\_\_Fungi;p\_\_unidentified;c\_\_unidentified;o\_\_unidentified;f\_\_unidentified;g\_\_unidentified | 0.2% | 0.0% | 1.0% | 0.0% | 0.0% |
