# Supplementary figures and images for: Foliar microbiome transplants confer disease resistance in a critically-endangered plant
Source: PeerJ. 2017 Nov 10;5:e4020. doi: 10.7717/peerj.4020 (PMC5683046; doi:10.7717/peerj.4020)

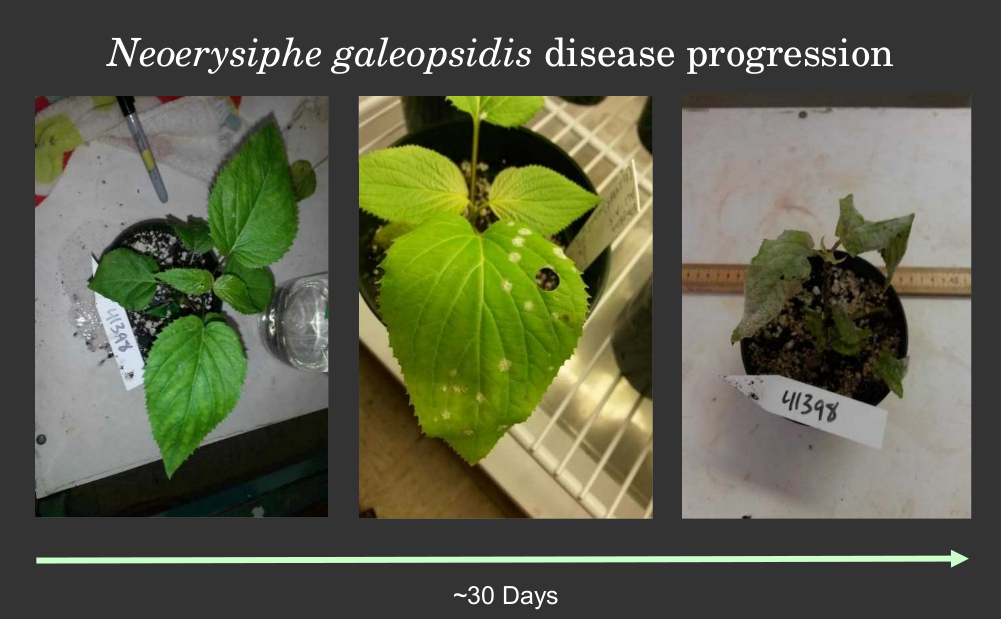

Supplement: Supplemental Information 3 [file peerj-05-4020-s003.png]

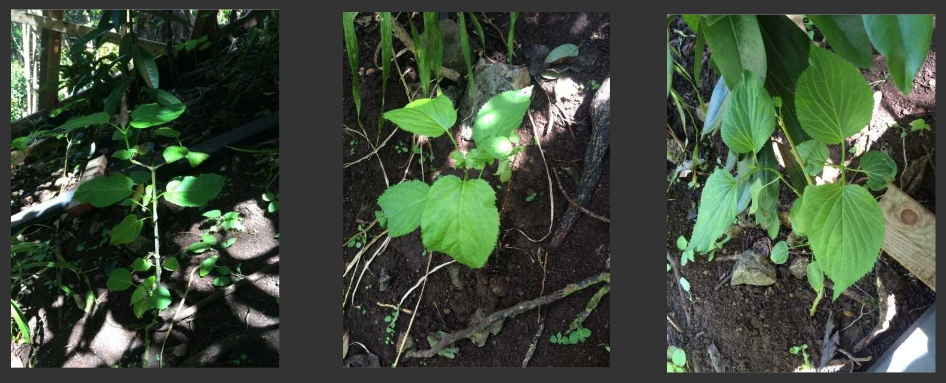

Supplement: Supplemental Information 4 [file peerj-05-4020-s004.png]

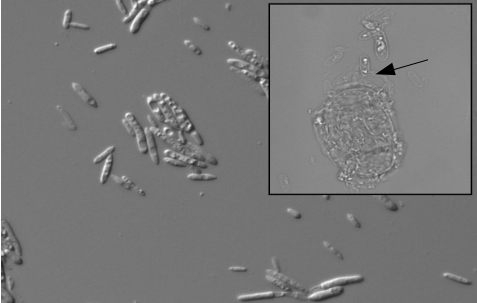

Supplement: Supplemental Information 5 — P. aphidis cell suspension, cultured from leaves of plant receiving the donor leaf slurry (DIC, 400 ×). Inset appears to show mycoparasitic activity of P. aphidis on an asexual N. galeopsidis spore (Brightfield, 1, 000 ×). [file peerj-05-4020-s005.png]
